# BMI mediates the causal relationship between depression and the risk of Rheumatoid arthritis: The results from NHANES 2003 to 2018 and the Mendelian studies

**DOI:** 10.1097/MD.0000000000043027

**Published:** 2025-09-12

**Authors:** Hongman Li, Peiting Li, Yusang Dai, Chun Zhao, Long Li

**Affiliations:** a Department of Hypertension, The Affiliated Hospital of Guizhou Medical University, Guiyang, Guizhou Province, China; b School of Clinical Medicine, Guizhou Medical University, Guiyang, Guizhou Province, China; c Department of Rheumatology and Immunology, The Affiliated Hospital of Guizhou Medical University, Guiyang, Guizhou Province, China; d Medical Examination Center, The Affiliated Hospital of Guizhou Medical University, Guiyang, Guizhou Province, China.

**Keywords:** body mass index, depression, Mendelian randomization analysis, National Health and Nutrition Examination Survey, rheumatoid arthritis

## Abstract

Depression and rheumatoid arthritis (RA) frequently coexist, leading to significantly impaired quality of life and increased healthcare costs. However, the causal relationship between these 2 conditions remains unclear. This study aimed to assess the causal association between depression and RA and to explore the mediating role of body mass index (BMI). We analyzed data from the National Health and Nutrition Examination Survey 2003 to 2018 using weighted multivariable-adjusted logistic regression to examine the association between depression and RA. Additionally, Mendelian randomization (MR) analyses, based on summary statistics from genome-wide association studies, were conducted to investigate the causal relationship. A 2-step MR analysis further evaluated the mediation effect of BMI. Observational analysis of National Health and Nutrition Examination Survey data showed a significant positive association between depression and RA risk (odds ratio [OR] = 1.25; 95% confidence interval [CI], 1.01–1.54). Higher depression scores were similarly associated with increased RA risk (OR = 1.02; 95% CI, 1.01–1.03). MR analysis provided genetic evidence supporting the causal link between depression and RA (OR = 4.61; 95% CI, 1.17–18.17). BMI partially mediated this causal relationship (mediation effect = 0.085), explaining 5.6% of the association. Our findings indicate a causal relationship between depression and an increased risk of RA, supported by both observational and genetic evidence. BMI plays a modest mediating role in this association.

## 1. Introduction

Rheumatoid arthritis (RA) is a prevalent autoimmune illness that causes inflammation throughout the body, particularly in the synovium. It affects around 0.5% to 1% of the world’s population.^[1]^ In addition to causing joint pain, swelling, and osteoporosis, severe cases may lead to disability.^[2]^ It has a substantial effect on the quality of life for patients and places a huge financial burden on both individuals and society.^[3]^ Research into the mechanisms and treatment methods for RA has made significant progress, but the specific mechanisms remain unclear, and a cure has not yet been achieved.^[4,5]^ Therefore, further investigation into the underlying mechanisms of RA is imperative to enhance strategies for its prevention and management.

Depression is a highly frequent mental condition, impacting more than 264 million individuals worldwide as of 2017.^[6]^ And the global prevalence of depression among older adults is 28.4%.^[7]^ According to the World Health Organization, severe depression was listed as the third most significant contributor to the world burden of disease in 2008. It is projected to surpass all other causes and become the primary contributor to the global burden of disease by 2030.^[8]^

Depression is an often-observed comorbidity in individuals with RA. According to Dan Pu research, almost 62% of patients with RA experience depression.^[9]^ Furthermore, there is increasing recognition that depression is linked to elevated disease activity and worse outcomes in RA.^[10]^ RA patients who experience depression have an 80% higher risk of all-cause mortality, in comparison to those who do not have depression.^[11]^ Therefore, determining the correlation between RA and depression could offer valuable insights for forthcoming screening and preventative guidelines.^[12]^ Current cross-sectional investigations have discovered a multifaceted reciprocal connection between depression and RA, but the results are contradictory. In addition, due to the confounding biases and reverse causation inherent in these observational studies, they are limited in establishing causal relationships and fail to fully elucidate the interplay between these conditions. Research on the molecular processes of RA and depression has identified shared immunological pathways and overlapping inflammatory cytokines, including IL-1, IL-18, and IL-6. These cytokines have both direct and indirect impacts on the brain.^[13,14]^ Nevertheless, the mechanisms remain incompletely comprehended. The lack of uniform viewpoints and the limits of observational research impede the ability to definitively determine the causal association between depression and RA. Therefore, further research is necessary.

As far as current knowledge extends, no research has definitively identified the probable mechanisms linking depression and RA. The correlation between Body mass index (BMI) and both depression and RA has been a subject of vigorous controversy. Research has indicated that there are reciprocal interactions between depression and BMI,^[15,16]^ and there is also evidence of a correlation between BMI and RA.^[17]^ Thus, we propose that BMI could serve as a mediator between depression and RA. This study commenced with a cross-sectional analysis utilizing a substantial and reliable sample from the NHAENS database. The objective was to assess the correlation between depression and the likelihood of developing RA. In addition, we utilized Mendelian randomization (MR) analysis, using single nucleotide polymorphisms (SNPs) as instrumental factors, to deduce the reciprocal causation between depression and RA from a genetic standpoint. To clarify the possible mechanistic pathways, we performed additional 2-step MR analyses to see if BMI acts as a mediator in this association.

## 2. Methods

### 2.1. Data source of NHAENS

The data were obtained from the publicly available analysis database NHAENS (https://www.cdc.gov/nchs/nhanes/?CDC_AAref_Val=https://www.cdc.gov/nchs/nhanes/index.htm), a widely utilized resource in current research. The National Health and Nutrition Examination Survey (NHANES) study protocol received approval from the Research Ethics Review Board of the National Center for Health Statistics, and all NHANES participants gave informed permission. The eligibility criteria for this study consisted of individuals who were enrolled at NHANES mobile examination centers between 2003 and 2018, were 20 years of age or older, reported having RA either through a questionnaire or oral communication, and underwent depression assessment using a health questionnaire to establish the presence of depression. The study sample comprised 9474 individuals that satisfied the criteria. The exclusion criteria were as follows: persons with incomplete data on RA (N = 24840); individuals with missing data on depression (N = 10476) (Fig. S1, Supplemental Digital Content, https://links.lww.com/MD/Q9).

### 2.2. Definition of RA and depression in NHAENS

The diagnosis of RA was acquired through a self-reported questionnaire survey (medical conditions: MCQ160a). More precisely, the participants were asked, “Have you ever been informed by a doctor or another healthcare professional that you have arthritis?” The possible responses include “Yes,” “No,” “Refused,” or “Don’t know.” An answer of “Yes” led to further questioning with the following options (MCQ190 [2003–2008], MCQ191 [2009–2010], MCQ195 [2011–2018]): “RA,” “Osteoarthritis,” “Psoriatic arthritis,” “Other,” “Refused,” or “Don’t know.” Research has demonstrated that there is an 85% agreement between individuals’ self-reported arthritis and the diagnosis made by medical professionals. This level of consistency makes it suitable for conducting investigations on a wide scale. The diagnosis of depression was evaluated with the Patient Health Questionnaire (PHQ-9) screening instrument. This survey comprises 9 questions, and according to prior studies, we employed a threshold of 10 points to ascertain the existence of depression.^[18]^ The diagnosis of depression was assessed using the PHQ-9 screening tool. This survey consists of 9 questions, and based on previous research, we used a threshold of 10 points to determine the presence of depression.^[19]^

### 2.3. Covariates in NHAENS

The covariates examined in this study encompass various demographic factors, including age (20–40, 41–65, >65), gender (male and female), race (Non-Hispanic White, Non-Hispanic Black, Other, Mexican American), marital status (yes or no), education level (less than high school, high school and above), alcohol consumption (whether the individual has consumed at least 12 drinks of any type of alcoholic beverage in any year), sleep disorders (SLQ, assessed by trained interviewers using the Computer-Assisted Personal Interview system at home to inquire about sleep time and wakefulness), and hypercholesterolemia (self-reported).

### 2.4. Data source of MR

The data of MR were sourced from Genome-wide association studies (GWAS) available at https://gwas.mrcieu.ac.uk/. Data for RA were obtained from a study recruiting 153,457 Europeans, including 6236 cases and 147,221 controls (id: finn-b-M13_RHEUMA). Data for depression were sourced from a large European cohort of 442,840 individuals (id: ukb-b-3822). BMI data were derived from a dataset comprising 532,396 samples from Europe (id: ebi-a-GCST90029007). All the original cohorts used in the study had received ethical approval and gained informed permission from the participants. The criterion for genome-wide significance was established at a *P*-value less than 5 × 10^−8^ to find genetic variations that are highly related to the exposure. In addition, SNPs that exhibited linkage disequilibrium (*r*^2^ <0.001, 10,000 kb) were removed from the analysis.

### 2.5. Data analysis

In the NHANES analysis, we employed multivariable-adjusted logistic regression to evaluate the correlation between depression scores, depression status, and RA. Three models were assessed to account for covariates: Model 1 was not adjusted; Model 2 included adjustments for age, sex, race, marital status, and education level; and Model 3 additionally accounted for age, sex, race, marital status, education level, obesity, alcohol consumption, and hypercholesterolemia. The findings were reported as odds ratios or β coefficients, along with their corresponding 95% confidence intervals (CI). Due to the intricate probability clustering design of NHANES, weights were considered in the statistical analysis of this study. The R software (version 4.0.2) was utilized for the 2-sample MR study of the chosen SNP data. A significance level of *P* <.05 was deemed to have statistical significance. The primary strategy used to evaluate the causal link between genetically predicted depression and the risk of RA was the inverse variance weighted (IVW) method. In addition, extra analyses were conducted using the weighted median and MR-Egger methods. Diverse techniques were utilized to evaluate the diversity and horizontal pleiotropy among SNPs. The IVW and MR-Egger methods were utilized to identify and handle outliers, while a random-effects model was employed to evaluate the stability of the results.

### 2.6. Mediation analysis

To examine the possible connections between depression and RA, we performed a TSMR analysis. This involved using multivariable MR analysis to investigate the role of BMI as a mediator. The TSMR analysis was conducted exclusively for SNPs that had substantial MR correlations in the original analysis. Initially, we employed the IVW technique in a 2-sample univariable MR analysis to get estimates of the overall impact of depression on the risk of developing RA (Beta_all), as well as the impact of depression on the risk of developing BMI (Beta1), separately. Afterwards, a multivariable MR analysis was performed to evaluate the influence of both depression and BMI on the chance of developing RA. This analysis provided an estimate of the effect of BMI on the risk of RA (Beta2). The multiplication of Beta1 and Beta2 was subsequently computed to evaluate the indirect effect (mediation effect) of BMI. The calculation of the proportion of mediation explained by BMI involved dividing the indirect effect by the total effect.

## 3. Result

### 3.1. Baseline characteristics

Table 1 presents the clinical and laboratory traits of the individuals involved in the study. The study comprised a total of 9474 participants, with females accounting for 60% and males accounting for 40% of the population. The population was predominantly composed of non-Hispanic White individuals, with the age range of 41 to 65 years being 54.3% of the total. RA was observed in 15% of the whole population, while depression was observed in 13%. RA patients were found to have a greater prevalence of depression based on their RA status. The depression scores were significantly elevated, indicating statistical significance. Furthermore, there were notable statistical disparities in age, education level, and total cholesterol (Table 1).

**Table 1:** Demographic and clinical characteristics of participants without RA and with RA.

Characteristic	Overall, N = 9474 (100%)^1^	No, N = 7662 (85%)*	Yes, N = 1812 (15%)*	*P*-value†
Depression score	4.2 (5.1)	4.1 (5.0)	4.8 (5.5)	**<.001**
Depression (%)				**.001**
No	8043 (87%)	6544 (88%)	1499 (84%)	–
Yes	1431 (13%)	1118 (12%)	313 (16%)	–
Age (%)				
20–40	783.0 (9.9%)	631.0 (9.5%)	152.0 (12.1%)	**.002**
41–65	4569.0 (53.4%)	3620.0 (53.0%)	949.0 (55.6%)	
>65	4122.0 (36.7%)	3411.0 (37.5%)	711.0 (32.3%)	
Gender (%)				
Male	3906 (40%)	3143 (40%)	763 (41%)	0.5
Female	5568 (60%)	4519 (60%)	1049 (59%)	
Race/ethnicity (%)				
Non-Hispanic White	4959 (77%)	4237 (79%)	722 (66%)	**<.001**
Non-Hispanic Black	2116 (10%)	1566 (9.0%)	550 (17%)	
Other	1396 (8.8%)	1112 (8.5%)	284 (11%)	
Mexican American	1003 (4.2%)	747 (3.7%)	256 (7.2%)	
Married/live with partner (%)				
No	4134 (37%)	3297 (36%)	837 (39%)	0.053
Yes	5340 (63%)	4365 (64%)	975 (61%)	
Education level (%)				
Below high school	2589 (18%)	2000 (17%)	589 (23%)	**<.001**
High School or above	6885 (82%)	5662 (83%)	1223 (77%)	
Obesity				
No	4729 (52%)	3824 (51%)	905 (52%)	0.7
Yes	4594 (48%)	3720 (49%)	874 (48%)	
Alcohol use (%)				
No	3031 (27%)	2411 (26%)	620 (30%)	.052
Yes	6290 (73%)	5127 (74%)	1163 (70%)	
Sleep disorder (%)				
No	7148 (73%)	5798 (73%)	1350 (72%)	.5
Yes	2311 (27%)	1853 (27%)	458 (28%)	
High cholesterol (%)				
No	4156 (47%)	3351 (47%)	805 (51%)	**.026**
Yes	4849 (53%)	3960 (53%)	889 (49%)	

Bold values indicate statistically significant differences with *P* < .001.

RA = rheumatoid arthritis, SD = standard deviation.

* Median (SD) for continuous; n (%) for categorical.

† Wilcoxon rank-sum test for complex survey samples; chi-squared test with Rao and Scott second-order correction.

### 3.2. Weighted logistic regression analysis of the association between depression and RA in NHANES

The findings from Table 2 indicate that there is a statistically significant relationship between higher depression scores (OR = 1.03 [95% CI, 1.01–1.04]) and the presence of depression (OR = 1.36 [95% CI, 1.14–1.63]) with an elevated risk of RA, with significant differences (*P* <.05). After adjusting for age, sex, ethnicity, marital status, and education level in Model II, both depression scores (OR = 1.02 [95% CI, 1.01–1.03]) and depression (OR = 1.23 [95% CI, 1.02–1.48]) continued to have a significant association with RA (*P* <.05). In Model III, even after controlling for age, sex, ethnicity, marital status, education level, obesity, alcohol consumption, and high cholesterol, there was still a significant association between RA and depression scores (OR = 1.02 [95% CI, 1.01–1.03]) as well as depression (OR = 1.25 [95% CI, 1.01–1.54]) (*P* <.05). These data suggest that there is a positive correlation between depression and the chance of having RA. Specifically, for every 1-unit rise in the depression score, the risk of RA increases by 2%. Additionally, person with depression have a 25% greater risk of developing RA compared to those without depression (Table 2).

**Table 2:** Weighted logistic regression analysis on the association between depression and rheumatoid arthritis.

	Model I OR (95% CI)	*P*-value	Model II OR (95% CI)	*P*-value	Model III OR (95% CI)	*P*-value
Depression score	1.03 (1.01–1.04)	<.001	1.02 (1.01–1.03)	.002	1.02 (1.01–1.03)	.003
Depression						
No	Reference		Reference		Reference	
Yes	1.36 (1.14–1.63)	.001	1.23 (1.02–1.48)	.031	1.25 (1.01–1.54)	.037
*P* for trend	.001	–	.012	–	.010	–

CI = confidence interval, OR = odds ratio.

Model I: None covariates were adjusted; Model II: Age, gender, race, marital status, education were adjusted; Model III: Age, gender, race, marital status, education, obesity, drink and high cholesterol were adjusted.

### 3.3. Subgroup analysis and interaction detection

Table 3 shows that depression is positively associated with RA among females, alcohol drinkers, and individuals aged over 65 years (*P* <.05). However, no interactions were observed between depression and age, sex, ethnicity, marital status, education level, alcohol consumption, sleep disorders, or high cholesterol levels (*P* for interaction >.05) (Table 3).

**Table 3:** Subgroup analysis.

Variable	OR	Lower	Upper	*P* for interaction
Depression	1.25	1.01	1.54	–
Age				
20–40	1.02	0.51	2.04	.516
41–65	1.16	0.89	1.5	
>65	1.58	1.06	2.37	
Gender				
Male	0.95	0.62	1.44	.118
Female	1.4	1.09	1.81	
Race				
Mexican American	1.13	0.74	1.72	.538
Non-Hispanic White	1.34	0.98	1.82	
Non-Hispanic Black	1.31	0.92	1.85	
Other	0.93	0.55	1.57	
Marital				
No	1.29	0.98	1.7	.927
Yes	1.2	0.89	1.62	
Education				
Below high school	1.2	0.89	1.64	.756
High School or above	1.24	0.94	1.65	
Drink				
No	1.11	0.77	1.6	.458
Yes	1.31	1.01	1.69	
Sleep disorder				
No	1.29	0.99	1.68	.722
Yes	1.21	0.84	1.74	
High cholesterol				
No	1.32	0.94	1.85	.840
Yes	1.17	0.88	1.55	

OR = odds ratio.

### 3.4. Causal relationship between depression and RA in the MR study

Based on the significant positive association identified between depression and RA in the multivariable regression analysis described previously, we conducted a bidirectional MR analysis to evaluate the causal link between depression and the increased risk of RA. As shown in Table 4, using the IVW method, the results indicate a positive causal relationship between depression and RA (OR = 4.61 [95% CI, 1.17–18.17], *P* = .029). Similar results were obtained using the weighted median method (OR = 8.12 [95% CI, 1.33–49.68], *P* = .023) and MR-Egger (OR = 0.11 [95% CI, 0.21–28.79], *P* = .733). Conversely, when conducting the reverse analysis of their relationship using the IVW method (detailed in Table S1, Supplemental Digital Content, https://links.lww.com/MD/Q10), the results indicate no significant association (OR = 1 [95% CI, 0.99–1], *P* = .53), suggesting that genetically, depression increases the risk of developing RA, but RA does not increase the risk of depression. Furthermore, no evidence of heterogeneity or horizontal pleiotropy was observed, as indicated by IVW (*P* = .860) and MR-Egger intercept (*P* = .560) (Table S2, Supplemental Digital Content, https://links.lww.com/MD/Q10 and Fig. S2, Supplemental Digital Content, https://links.lww.com/MD/Q9).

**Table 4:** Mendelian randomization estimates for the association between genetically predicted depression and rheumatoid arthritis.

Method	SNPs	B	SE	*P*-value	OR_95_CI	*P*-value for MR-Egger intercept	*P*-value for heterogeneity test
IVW	13	1.53	0.7	.029	4.61 (1.17–18.17)	–	.86
Weighted median	13	2.09	0.92	.023	8.12 (1.33–49.68)	–	–
MR-Egger	13	−2.17	6.19	.733	0.11 (0.21–28.79)	0.56	–

CI = confidence interval, IVW = Inverse variance weighted, MR = Mendelian randomization, OR = odds ratio, SNPs = single nucleotide polymorphisms.

### 3.5. Mediation analysis results

**Figure 1.**
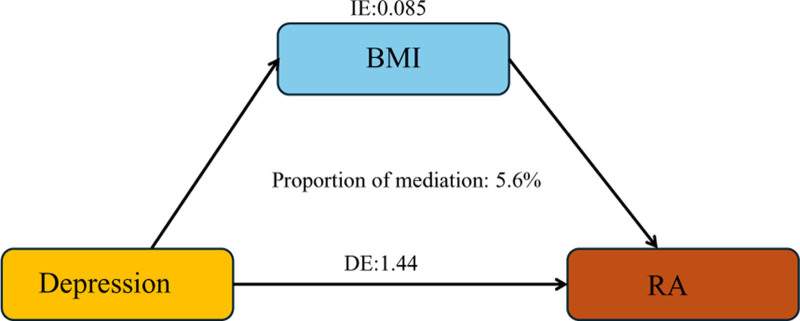
Mediate effects in the study.

Univariable MR analysis provides causal estimates that indicate the overall effect of the exposure on the outcome. In contrast, multivariable MR analysis can estimate the direct causal effect of the exposure on the outcome by considering potential mediators. The difference between the causal estimates from univariable (total effect) and multivariable MR analysis (direct causal effect) implies that the causal effect operates at least partially through potential mediators (indirect effect).^[20,21]^ Put simply, employing multivariable MR mediation analysis allows us to calculate the extent to which BMI mediates the relationship between depression and RA.^[22]^ The results of our 2-step MR mediation study demonstrate that BMI mediates the causal connection between depression and RA, with a mediation effect of 0.085. This accounts for 5.6% of the total effect. The findings indicate that BMI acts as a partial mediator in the causative association between depression and RA (Fig. 1 and Supplementary Text 1, Supplemental Digital Content, https://links.lww.com/MD/Q11).

## 4. Discussion

This study utilized cross-sectional analysis from the NHANES 2003 to 2018 cohort and MR 2-sample analysis to examine the causal relationship between depression and RA. Additionally, we investigated the potential effect of BMI as a mediator in this association. The findings of our cross-sectional investigation revealed a greater prevalence of depression and elevated depression scores among patients with RA. Multivariable logistic regression analysis showed a significant positive correlation between depression and RA after adjusting for potential confounders, consistent with most previous studies. However, as cross-sectional studies are prone to confusing bias and reverse causal relations, previous studies were unable to establish a causal relationship between depression and RA. Therefore, we employed 3 different estimation methods in MR analysis to address these limitations. The results consistently confirmed that depression significantly increased the incidence of RA, without any evidence of a reverse relationship. Furthermore, utilizing TSMR analysis, we confirmed that BMI played a certain mediating role in this relationship. This work is the first to discover a factor that mediates the connection between the risk of depression and RA using MR mediation analysis.

The relationship between depression and RA has been a subject of sustained interest. Depression is commonly observed in individuals with RA, with the prevalence of depression in RA being 2 to 3 times higher than in the general population.^[23]^ Interestingly, current cross-sectional investigations have found a bidirectional relationship between them, especially in the elderly population^11^. Similarly, a comprehensive retrospective study conducted by Huang et al in South Korea involved a total of 38,487 patients with RA and 192,435 people in the control group. The participants were followed for an average of 4.1 years. A total of 6422 people with RA and 20,641 people in the control group experienced the onset of depression. The participants with RA had a 1.66-fold increased risk of getting depression compared to the control group (adjusted hazard ratio [aHR] 1.66 [95% CI, 1.61–1.71]).^[24]^ Furthermore, a meta-analysis conducted by Ng CYH et al evaluated the bidirectional relationship between RA and depression. The research included 11 cohort studies, with a combined sample size of 39,130 RA patients, 550,782 depression patients, and 7802,230 individuals in the control group. The findings indicated that, in comparison to the control group, individuals with RA exhibited a 47% elevated susceptibility to acquiring depression. Conversely, patients with depression had a 34% increased likelihood of developing RA. These results demonstrate a bidirectional relationship between them.^[11]^ The reasons behind this relationship include the psychological burden caused by pain, disability, or fear of disability resulting from RA, as well as the economic burden, leading to depression.^[25]^ On the other hand, RA patients’ pain scores may also be exacerbated by non-inflammatory pain secondary to depression, anxiety, sleep disorders, and other factors.^[26]^ Nevertheless, it is important to acknowledge that all of this research examining the correlation between RA and depression is cross-sectional in nature. The current study has identified shared inflammatory cytokines as a biological link between depression and RA. Several studies have examined and discovered increased levels of pro-inflammatory cytokines TNF-α, IL-1, and IL-6 in individuals diagnosed with depression.^[27–29]^ Additionally, it is believed that immune-mediated inflammation can affect the central nervous system through neuronal and humoral pathways.^[30]^ A study conducted on animal models discovered that the increase in peripheral inflammatory cytokines IL-1 and TNF-α triggers the activation of serotonin transporter through the p38 MAPK pathway, resulting in the development of depression.^[31]^ However, the causal relationship between the 2 remains uncertain. Therefore, our MR method elucidates the causal relationship between depression and RA from a genetic perspective. Previously, a MR analysis by Sijia Fang et al did not discover any indication of a causal relationship between depression and RA,^[32]^ which is inconsistent with our results. Additionally, another Mendelian study on the mediation analysis of depression and RA found that 28 inflammatory factors were not the mechanism of their comorbidity.^[33]^ Currently, there is a lack of research into the mechanisms of mediation between them. Through the MR analysis of the European population, we have a more comprehensive understanding of the direct and beneficial effects of depression on RA. Depression promotes the occurrence of RA, and through the 2-step MR method, we found that BMI mediates their causal effect, with a mediation proportion of 5.6%. Furthermore, the correlation between depression and BMI has been a subject of contentious discussion for a considerable period. Studies indicate a reciprocal relationship between depression and BMI.^[15,16]^ The primary factors involve negative emotions stimulating the release of glucocorticoids and insulin, resulting in heightened appetite. Furthermore, a majority of individuals suffering from depression experience sleep disturbances and decreased levels of physical activity, which can contribute to the development of obesity.^[34]^ Leptin, disruption of the gut microbiota, and inflammatory signaling pathways serve as connecting factors in this process.^[15]^ On the other hand, negative emotions about body dissatisfaction in obese patients can lead to depression.^[35]^ There is now more evidence supporting the notion that BMI can lead to depression. However, research results on the impact of depression on future BMI are inconsistent and subject to debate.^[36,37]^ In future research, it would be beneficial to explore the potential influence of additional metabolic indicators on the relationship between depression and RA. Moreover, there is still no agreement on the correlation between BMI and the onset of RA. A meta-analysis study revealed that there is an elevated risk of getting RA in populations with obesity. Adipose tissue can contribute to the onset of RA by generating inflammatory substances such as TNF-α, IL-6, and leptin.^[38,39]^ Therefore, from the perspective of this study, enhancing the screening of RA in patients with depression and advocating for a healthy diet and physical exercise to control BMI in depression patients will help prevent the occurrence and development of RA. Although our MR mediation analysis identified BMI as a statistically significant mediator in the relationship between depression and RA, it is important to acknowledge that the mediation proportion was modest (5.6%). This indicates that BMI may only partially explain the mechanistic pathway, and other factors likely contribute to the overall effect. These pro-inflammatory mediators could represent additional potential mediators linking the 2 conditions.^[40,41]^ Future studies incorporating cytokine profiles and broader metabolic indicators would be valuable in providing a more comprehensive understanding of the underlying biological mechanisms.

In addition, it is important to acknowledge several limitations of this study. Firstly, the diagnosis of RA was obtained through verbal reporting, which may introduce recall bias. Secondly, the data were sourced from the European population, which may restrict the generalizability of the findings. Thirdly, the exclusion of many individuals without data on RA and depression could present a potential selection bias. Fourthly, even after accounting for numerous confounding factors, there may be other unmeasured confounders influencing the results.

Overall, by integrating NHANES with large amounts of observational data and MR analyses, we have demonstrated the causal relationship between depression and RA. Moreover, we assessed the role of BMI as a mediator in this relationship, providing new insights into the cause-and-effect relationship between depression and RA. Routine screening for RA is necessary for individuals with depression to facilitate early implementation of intervention measures before the onset of RA and its associated complications. However, our findings need further validation, and additional elucidation of their potential mechanisms is required.

## Acknowledgments

The authors thank the National Health and Nutrition Examination Survey (NHANES) data and GWAS data.

## Author contributions

**Conceptualization:** Hongman Li.

**Formal analysis:** Hongman Li, Peiting Li, Chun Zhao, Yusang Dai.

**Methodology:** Hongman Li, Peiting Li.

**Supervision:** Long Li.

**Writing – original draft:** Hongman Li.

**Writing – review & editing:** Chun Zhao, Yusang Dai, Long Li.

## Supplementary Material


