# Supplementary figures and images for: BMI mediates the causal relationship between depression and the risk of Rheumatoid arthritis: The results from NHANES 2003 to 2018 and the Mendelian studies
Source: Medicine (Baltimore). 2025 Sep 12;104(37):e43027. doi: 10.1097/MD.0000000000043027 (PMC12440480; doi:10.1097/MD.0000000000043027)

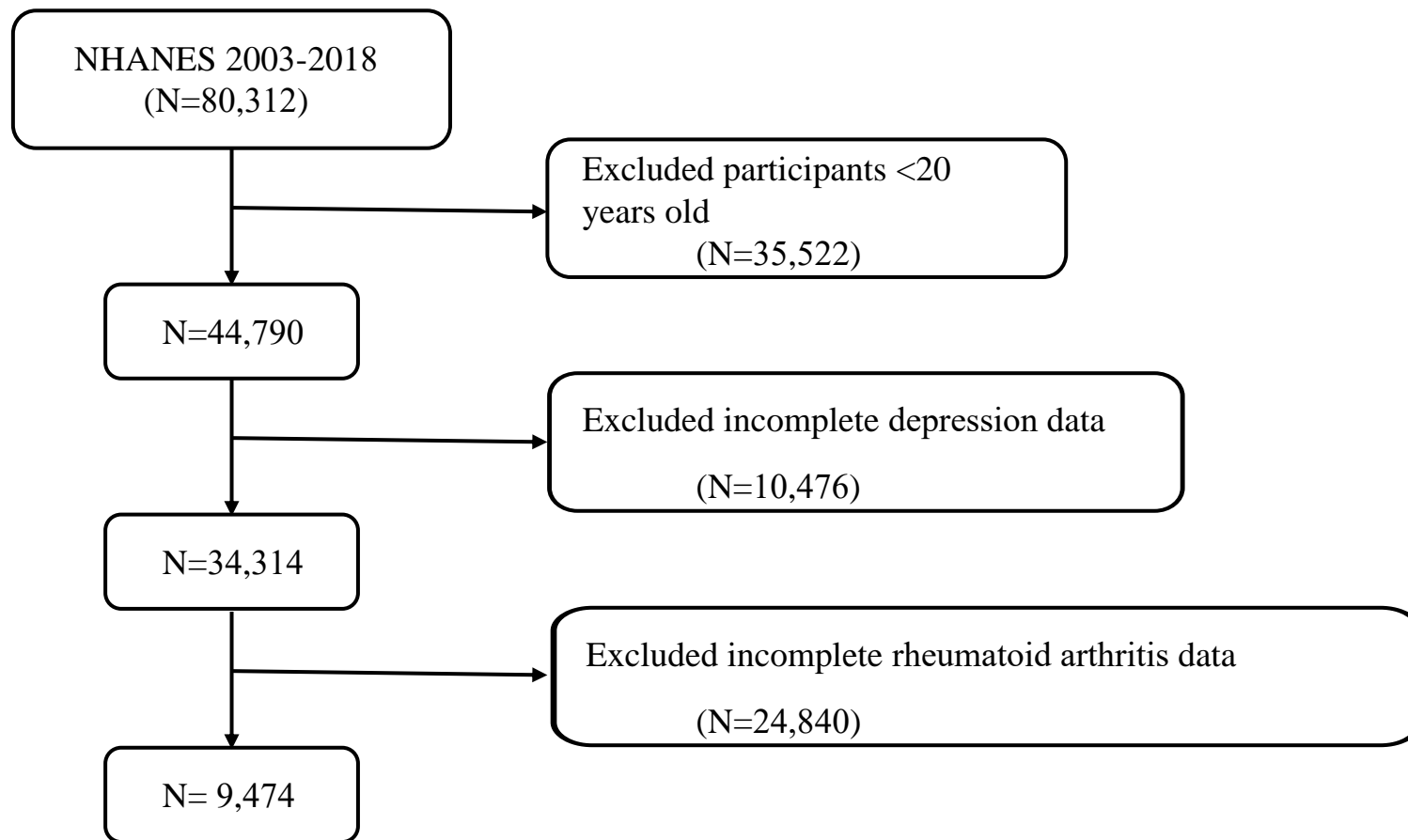

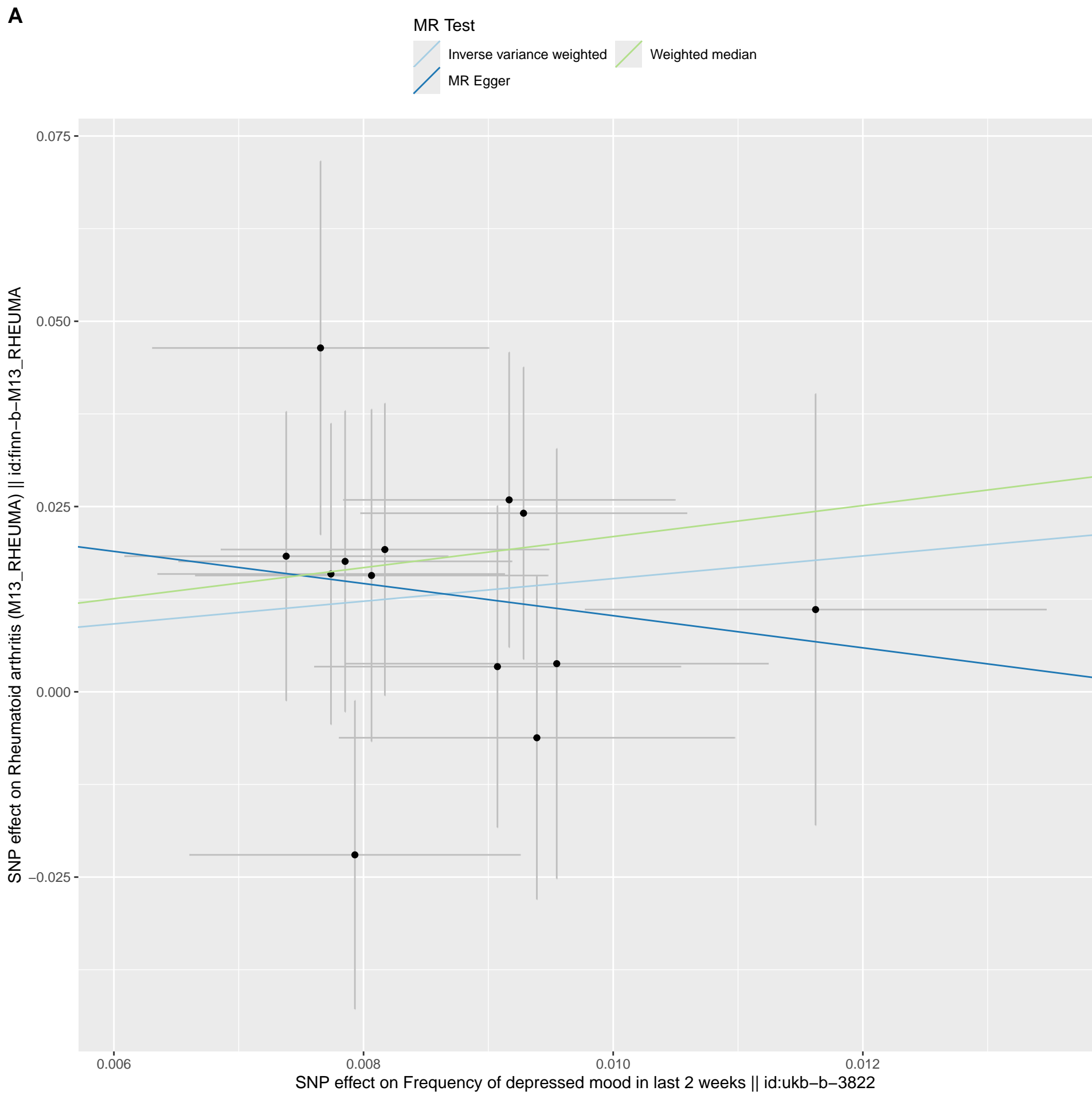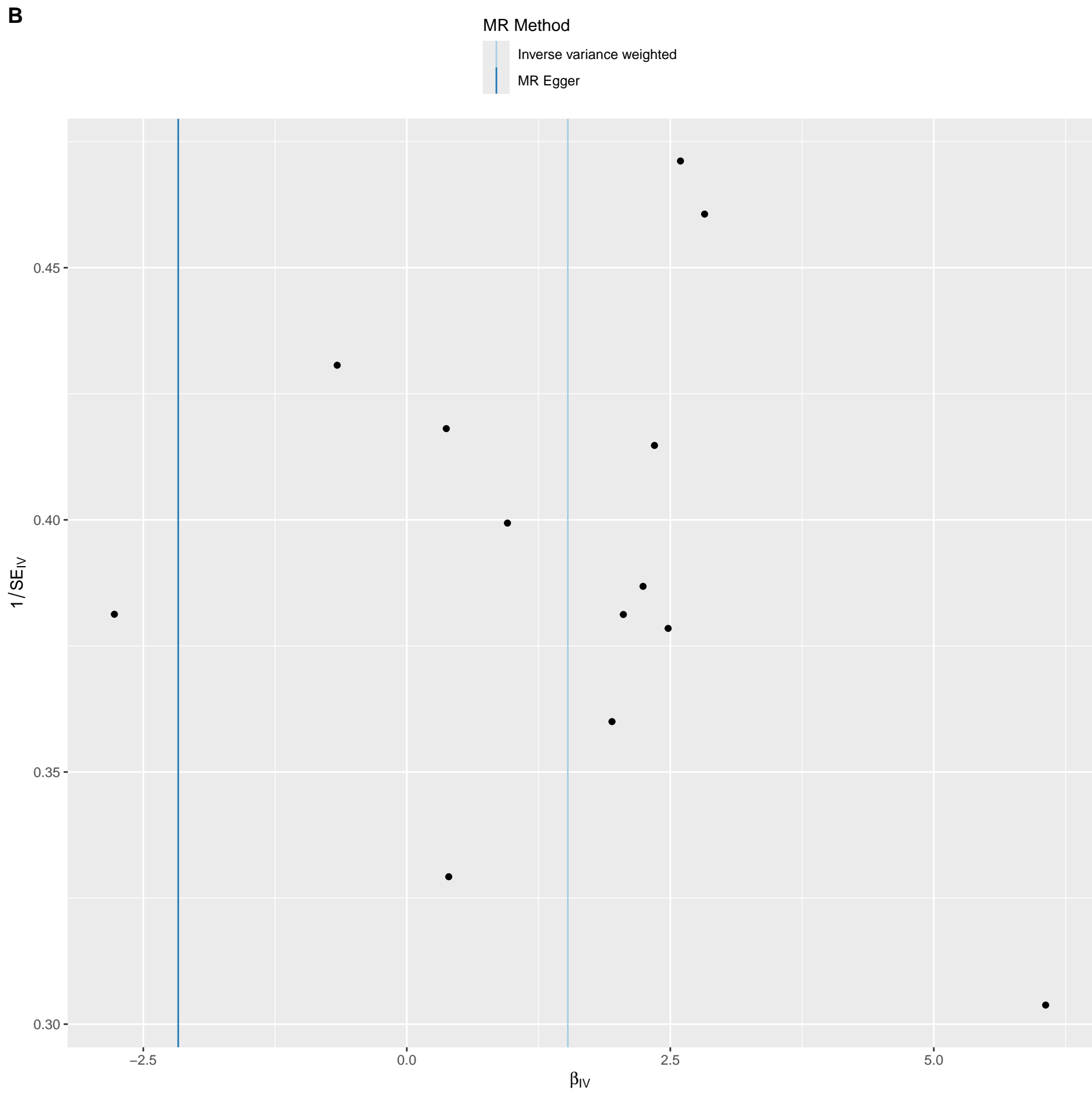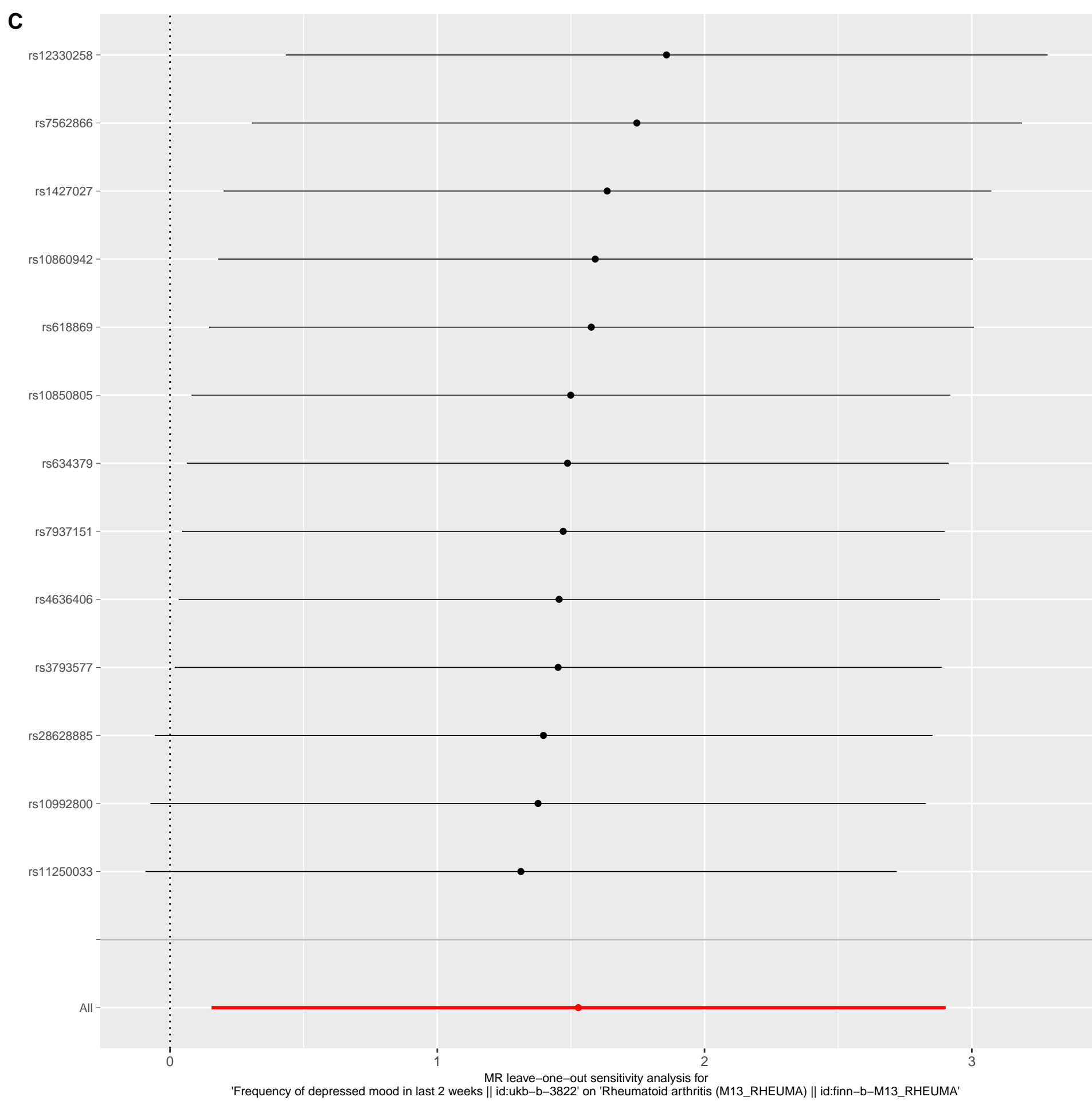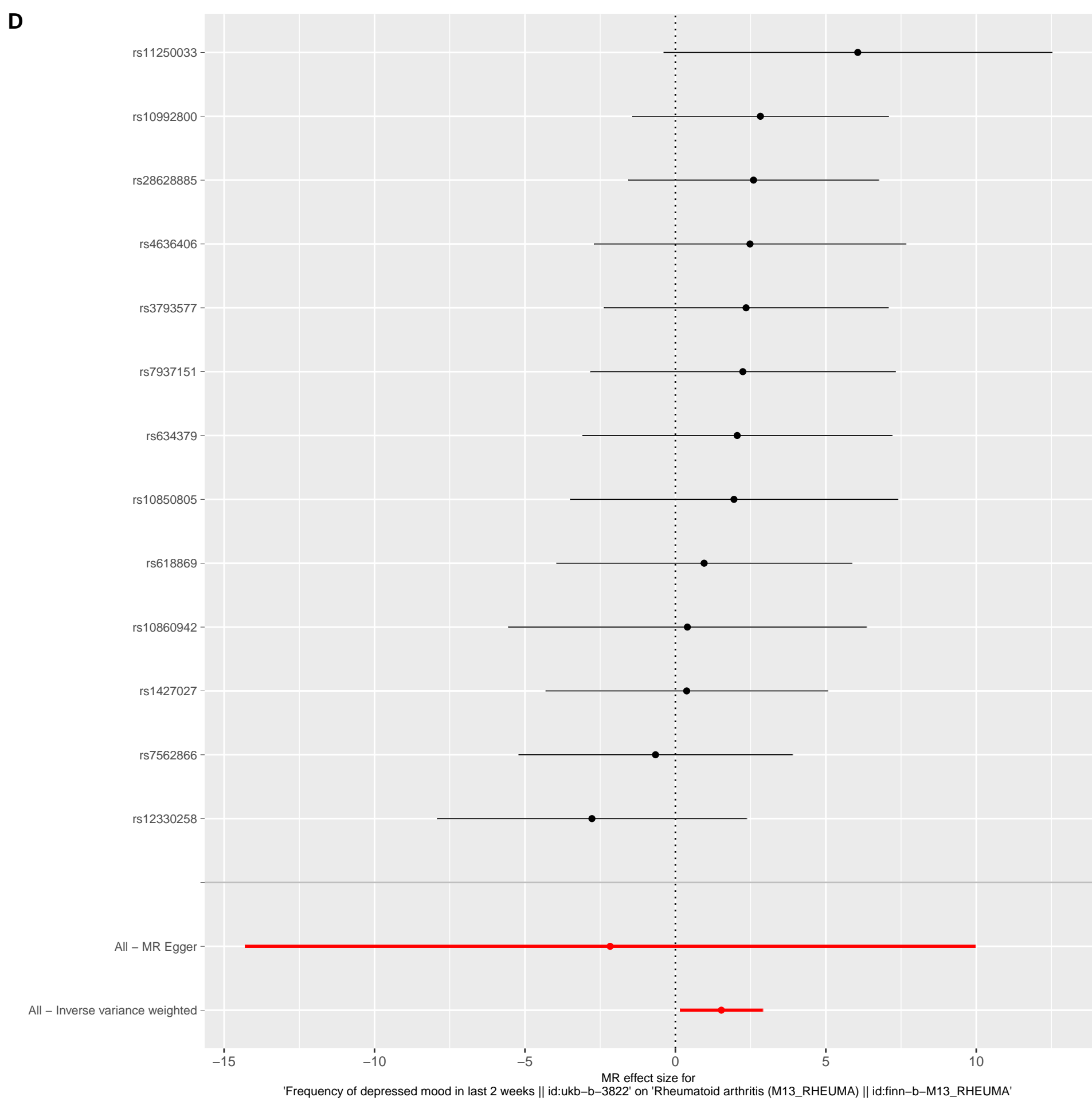

Supplement: Supplementary file 1 [file medi-104-e43027-s001.pdf]
